# The Role of Stress and Trauma in the Onset of Mood Disorders

**DOI:** 10.1192/j.eurpsy.2025.1830

**Published:** 2025-08-26

**Authors:** D. Jelaga, B. Mihaela, O. Valentin

**Affiliations:** 1 Department of Mental Health, Medical Psychology, and Psychotherapy, Nicolae Testemițanu State University of Medicine and Pharmacy of the Republic of Moldova, Chişinǎu, Moldova, Republic of

## Abstract

**Introduction:**

Stressful experiences and traumatic events are major contributors to the development of mood disorders, which affect about 8% of the global population. The interaction between stress, trauma, and mood disorders is multifaceted, involving neurobiological, psychological, and social factors. This study aims to analyze the prevalence, gender differences, and neurophysiological changes linked to these conditions, highlighting the importance of timely interventions for prevention and treatment.

**Objectives:**

To investigate how chronic stress and trauma contribute to the development of mood disorders, examine the impact of resilience factors, and explore the associated neurochemical and structural brain changes.

**Methods:**

A review of literature was performed using psychiatric textbooks, clinical guidelines, and databases such as PubMed/MEDLINE, NCBI, PsycINFO, and Google Scholar. The analysis focused on studies published between 2016 and 2023, with search terms including “chronic stress”, “traum”, “mood disorders” and “resilience”.

**Results:**

An analysis of 30 studies revealed that 65% of individuals exposed to prolonged stress experience mood disorders. Trauma survivors have increased risk of developing depression, women 35% more likely to suffer from depression than men. In contrast, men show a 25% higher incidence of developing bipolar disorder following trauma. Resilience factors such as family support and psychological counseling can reduce the risk of mood disorders by 20%. Additionally, 35% of patients with mood disorders exhibit comorbidities such as PTSD or substance use disorders. Neurochemical changes include a 50% reduction in dopamine levels, while 40% of individuals show hippocampal atrophy linked to chronic stress.

**Conclusions:**

Chronic stress and trauma are key factors in the onset of mood disorders, with distinct gender differences and significant neurobiological changes. Early intervention, focusing on resilience enhancement and psychosocial support, can reduce the long-term effects of stress and trauma, improving mental health outcomes.

**Disclosure of Interest:**

None Declared

